# The Children's Eating Behaviour Questionnaire: factorial validity and association with Body Mass Index in Dutch children aged 6–7

**DOI:** 10.1186/1479-5868-5-49

**Published:** 2008-10-20

**Authors:** Ester FC Sleddens, Stef PJ Kremers, Carel Thijs

**Affiliations:** 1 Department of Health Education and Promotion, Maastricht University, PO Box 616, 6200 MD, Maastricht, the Netherlands; 2 Department of Epidemiology, School of Public Health and Primary Care (Caphri), Maastricht, the Netherlands; 3 Nutrition and Toxicology Research Institute Maastricht (NUTRIM), Maastricht University, PO Box 616, 6200 MD, Maastricht, the Netherlands

## Abstract

**Background:**

The Children's Eating Behaviour Questionnaire (CEBQ) is a parent-report measure designed to assess variation in eating style among children. In the present study we translated the CEBQ and examined its factor structure in a sample of parents of 6- and 7-year-old children in the Netherlands. Additionally, associations between the mean scale scores of the instrument and children's body mass index (BMI) were assessed.

**Methods:**

In total, 135 parents of primary school children aged 6 and 7 completed the questionnaire (response rate 41.9%). Children's BMI was converted into standardised z-scores, adjusted for child gender and age to examine the association between mean scale scores and child weight status.

**Results:**

Results generally confirmed the theoretical factor structure, with acceptable internal reliability and between-subscale correlations. Linear regression analyses revealed that BMI z-scores were positively associated with the 'food approach' subscales of the CEBQ (food responsiveness, enjoyment of food, emotional overeating) (β's 0.15 to 0.22) and negatively with 'food avoidant' subscales (satiety responsiveness, slowness in eating, emotional undereating, and food fussiness) (β's -0.09 to -0.25). Significant relations with child BMI z-scores were found for food responsiveness (p = 0.02), enjoyment of food (p = 0.03), satiety responsiveness (p = 0.01) and slowness in eating (p = 0.01).

**Conclusion:**

The results support the use of the CEBQ as a psychometrically sound tool for assessing children's eating behaviours in Dutch children and the study demonstrates its applicability in overweight-related studies.

## Background

Especially during the last few decades the prevalence rates of childhood overweight and obesity have reached epidemic proportions worldwide [1], and also in the Netherlands [2]. Obese children face difficulties in their social life and run a substantially increased risk of becoming our future generation of obese, chronically diseased adolescents and adults [3,4]. Despite widely held beliefs regarding the importance of factors promoting excessive weight gain in children, it still remains a challenge to discover the underlying child behaviours that might contribute to differences in weight status across children [5-7]. Unravelling these factors will inform the development of evidence-based intervention programs to prevent overweight and obesity in children.

In the past, a number of psychometric instruments have been developed to assess eating behaviour in children, including the Children's Eating Behaviour Questionnaire (CEBQ) [7], the Dutch Eating Behaviour Questionnaire (DEBQ) [8,9], the Children's Eating Behavior Inventory (CEBI) [10] and the BATMAN (Bob and Tom's Method of Assessing Nutrition) [11]. The CEBQ is generally regarded as one of the most comprehensive instruments in assessing children's eating behaviour. The instrument was developed and validated in the United Kingdom, and recently the instrument has been validated in a Portuguese sample [6]. To our knowledge, no other validation studies have been performed on the CEBQ, but the instrument has been used for different research purposes, e.g., to examine associations with child body mass index (BMI) [6,12,13]; to compare appetite preferences in children of lean and obese parents [12,14]; to discover continuity and stability in children's eating behaviours across time [15]; and to examine eating behaviours of children with idiopathic short stature [16].

The CEBQ consists of the following eight scales. The scales food responsiveness (FR) and enjoyment of food (EF) reflect eating in response to environmental food cues. In response to these cues appetitive responses and eating rate have been found to strongly increase in overweight or obese children [5,7,13]. The scale desire to drink (DD) reflects the desire of children to have drinks to carry around with them, usually sugar-sweetened drinks [7]. Several studies found that BMI was positively associated with frequent consumption of sugar-sweetened drinks [17,18] and a decline in soft drink consumption would result in a reduction of overweight and obese children [19]. Satiety responsiveness (SR) represents the ability of a child to reduce food intake after eating to regulate its energy intake. Infants tend to be highly responsive to internal hunger and satiety cues, whereas this level of responsiveness decreases with advancing age [5,13,20]. Thus, during childhood, children will gradually lose the ability to effectively self-regulate energy intake, thereby promoting episodes of over-consumption and subsequently excessive weight gain. High scores on the scale slowness in eating (SE) is characterised by a reduction in eating rate as a consequence of lack of enjoyment and interest in food. Compared to their leaner counterparts, obese children have an increased consumption and have less reduction of their eating rate during the end of a meal [21]. Food fussiness (FF) is usually defined as rejection of a substantial amount of familiar foods as well as 'new' foods, thereby leading to the consumption of an inadequate variety of foods [22]. This type of eating style is characterised by a lack of interest in food [23], and slowness in eating [24]. Conflicting findings regarding the relationship between fussy eating and BMI in children have been found [23,25-27]. The scales emotional overeating (EOE) and emotional undereating (EUE) can be characterised by either an increase or a decrease in eating in response to a range of negative emotions, such as anger and anxiety. Emotional overeating has been found to be positively related to child BMI, whereas emotional undereating was negatively related to child BMI [6,28].

The original CEBQ scale has been shown to have good internal consistency (Cronbach's alphas ranging from 0.72 to 0.91) [7], adequate two-week test-retest reliability (correlation coefficients ranging from 0.52 to 0.87) [7] and construct validity [5]. Principal Components Analyses showed that each scale had a single factor, which explained 50–84% of the variance, and an overall factor analysis resulted in a verification of the hypothesised (theoretical) scales [7].

The present study aimed to examine the factorial nature of the CEBQ in a Dutch sample of 6- and 7-year-old children. Specific objectives were to translate the CEBQ into the Dutch language, to assess its psychometric properties and to compare them with the original CEBQ, and to demonstrate its application in overweight-related studies by examining its association with the child's BMI. We hypothesised that overweight and obese children would have higher scores on 'food approach' subscales (i.e. FR, EF, EOE) and lower scores on 'food avoidant' subscales (i.e. SR, SE, EUE, FF) of the CEBQ.

## Methods

### Overview of procedures and participants

In total, 334 questionnaires were distributed among parents with the Dutch nationality by teachers of third graders (6- to 7-year-olds) of seven primary schools in Maastricht and surroundings, the Netherlands. Overall, 140 completed questionnaires were returned (41.9%). The response rate per school ranged from 15.0% to 60.7%. Five children were excluded, because the parents did not have the Dutch nationality. The mean age of the participating children was 6.5 years (standard deviation 0.5), consisting of two approximately equal-sized age groups: 6-year-old children (*N *= 71), and 7-year-old children (*N *= 62), two cases with no age indicated. Gender was equally divided across our sample, girls (*N *= 67) and boys (*N *= 68). With respect to parental education, seven levels were distinguished. A total of 24 parents (9.2%) completed lower general secondary education as highest educational level (N_father _= 12; 9.4%, N_mother _= 12; 9.0%). Other educational levels that were distinguished (in ascending order) were intermediate general secondary education (N_father _= 7; 5.5%, N_mother _= 6; 4.5%), intermediate vocational education ((N_father _= 36; 28.1%, N_mother _= 45; 33.6%), intermediate/high general secondary education (N_father _= 9; 7.0%, N_mother _= 10; 7.5%), higher general secondary education (N_father _= 1; 0.8%, N_mother _= 1; 0.7%), higher vocational education, college (N_father _= 37; 28.9%, N_mother _= 43; 32.1%), and higher vocational education, university (N_father _= 26; 20.3%, N_mother _= 17; 12.7%).

### Measures

The CEBQ was translated into Dutch by a team of four experts on eating behaviour at Maastricht University (the Netherlands) who are Dutch native speakers and fluent speakers of the English language (the two authors of this manuscript ES and SK, and two colleagues of the Department of Health Education and Promotion). Translations were cross-checked by this team and in case of inconsistencies between the translations, team meetings were held to discuss the particular item; for some issues, we contacted the developer of the instrument (Prof. Wardle) [7]. All translators approved the final translation.

The CEBQ consists of 35 items comprising eight subscales, each containing 3 to 6 items. Parents are asked to rate their child's eating behaviour on a five-point Likert scale (never, rarely, sometimes, often, always; 1–5). Sample scale items include for example 'Given the choice, my child would eat most of the time', and 'My child leaves food on his/her plate at the end of a meal'. In table 1, all items of the CEBQ are displayed.

### Body Mass Index

Parents were asked to report their children's height and weight to calculate BMI. Specific age and gender BMI cut-off points were used to define underweight [29] and overweight/obesity [30]. Additionally, a child's BMI was converted to a standardised z-score, adjusting for age and gender, based on reference data of the Fourth Dutch National Growth Study (1997) [31]. Parental reported weight and height of their children was available for 115 (85.2%) respondents.

### Statistical procedures

A Principal Components Analysis (PCA) with Varimax rotation was performed on all items of the CEBQ to determine if the original eight-factor structure (CEBQ) [7] would be replicated in our sample.

Both internal reliability coefficients (Cronbach's alphas) and (average) corrected item-total correlations were calculated. Guidelines exist to interpret (average) corrected item-total correlations, which correct for the contribution of the items to the scale. For the present study, we used the guidelines by Nunnally, who considered that correlations above 0.30 are 'good' and correlations below 0.15 may be unreliable (i.e. because they are wrongly interpreted by the study participants and/or are do not measure the same construct as the subscale) [32]. The reliability estimates were compared with those found by previous validation studies [6,7].

Pearson's correlations were computed to evaluate relationships between mean item scale scores on each of the eight factors of the CEBQ originally found by Wardle et al. [7]. Interpretations were based on Cohen's descriptive guidelines [33], correlations between 0.5 and 1.0 being considered as large, correlations between 0.3 and 0.5 as medium, and correlations between 0.1 and 0.3 as small.

Gender and age differences between scores were calculated using independent samples t-tests. A series of multiple linear regression analyses was conducted to examine associations between scores on the subscales of the CEBQ with children's BMI z-scores as the dependent variable. Every subscale of the questionnaire was entered into the analysis separately with the following covariables to correct for potential confounding: child's gender and age; parental education, ranging from 1 (lowest level of education) to 7 (highest level of education); and parental employment status, dichotomised into 1 (employed) and 2 (non-employed). Missing anthropometric data was present for 20 children, and therefore BMI z-scores of these children could not be calculated. Those missing BMI z-scores were replaced using the mean imputation method. The sample size of the current study (*N *= 135) enables the detection of an additional explained variance of 6% (ΔR^2 ^= .06) in the prediction of one unit change in BMI z-score, with a power of .80 (alpha .05). In addition, one-way analysis of variance for comparison by weight status was used to examine differences in scale scores by child BMI groups and to assess the possibility of a non-linear relationship between BMI and eating style constructs. BMI was categorised into three weight categories, underweight (*N = *20; 17.4%), normal weight (*N = *83; 72.2%), and overweight/obesity (*N = *12; 10.4%; 10 overweight and 2 obese children grouped together to increase the statistical power).

## Results

### Factor analysis

The factor analysis revealed a seven-factor solution, presented in table 1. The seven factors accounted for 62.8% of the total variance. The items from two scales (EOE and FR) loaded onto the same factor, which we propose to name 'overeating' (table 2). Most of the scale items loaded as expected and their factor loadings were comparable to those obtained in the original study by Wardle et al. [7] and the study by Viana et al. [6]. However, four items deserve special attention. First of all, the item 'my child is always asking for food' did not load onto the expected factor FR, but on EF. Second, the item 'my child eats more when annoyed' loaded most highly onto the EUE factor (.55), but has been retained on the EOE scale on theoretical grounds (factor loading .47). The item 'my child eats more and more slowly during the course of a meal' loaded most highly onto the SR factor (.63), but has been retained on the SE factor (.39). Separate Principal Components Analyses (PCAs) on the seven final scales showed that six of them constituted a single factor with an eigenvalue greater than one, accounting for 51–70% of the variance across the scales. One exception was the overeating scale, which had two factors with an eigenvalue greater than one (revealing the original FR and EOE scales), accounting for 42% of the variance across the seven scales. In spite of our seven-factor solution, we performed further statistical analyses on the eight subscales as defined by Wardle and colleagues [7], in order to allow comparison with the original subscales and in line with the previous Portuguese study [6].

**Table 1:** Factor loadings on Varimax Rotated Solution of Principal Components Analysis (CEBQ, *N *= 135)

Scale name and items	Loading	Scale name and items	Loading
**Food fussiness (Factor 1; 13.2% variance)**		**Satiety responsiveness (Factor 4; 8.8% variance)**	
My child refuses new foods at first	.83	My child has a big appetite	.32
My chid enjoys tasting new foods	.87	My child leaves food on his/her plate at the end of a meal	.69
My child enjoys a wide variety of foods	.77	My child gets full before his/her meal is finished	.70
My child is difficult to please with meals^(e)^	.56	My child gets full up easily	.65
My child is interested in tasting food s/he hasn't tasted before	.88	My child cannot eat a meal if s/he has had a snack just before	.55
My child decides that s/he doesn't like food, even without tasting it	.75		
		**Emotional undereating (Factor 5; 8.7% variance)**	
**Enjoyment of food (Factor 2; 10.5% variance)**		My chid eats less when s/he is angry	.78
My child loves food	.69	My child eats less when s/he is tired	.77
My child is interested in food	.66	My child eats more when s/he is happy	.71
My child is always asking for food^(b)^	.53	My child eats less when s/he is upset	.72
My child enjoys eating	.62		
My child looks forward to mealtimes	.56	**Desire to drink (Factor 6; 6.3% variance)**	
		My child is always asking for a drink	.74
**Food responsiveness/Emotional overeating^(a) ^(Factor 3; 9.3% variance)**		If given the chance, my child would drink continuously throughout the day	.83
My child eats more when worried	.43	If given the chance, my child would always be having a drink	.81
My child eats more when annoyed^(c)^	.47		
If allowed to, my child would eat too much	.73	**Slowness in eating (Factor 7; 6.0% variance)**	
My child eats more when anxious	.61	My child finishes his/her meal very quickly	.66
Given the choice, my child would eat most of the time	.65	My child eats slowly	.71
My child eats more when s/he has nothing else to do	.67	My child takes more than 30 minutes to finish a meal	.51
Even if my child is full up, s/he finds room to eat his/her favourite food	.38	My child eats more and more slowly during the course of a meal^(d)^	.39
If given the chance, my child would always have food in his/her mouth	.72		

^(a) ^FR and EOE loaded onto the same factor in the final solution, so one scale was developed which we propose to name 'overeating' (OE).

^(b) ^The item 'My child is always asking for food' loaded most highly onto the EF factor (.53) than on the FR factor (.05), where the factor originally belongs. Therefore, this item was incorporated in the factor EF.

^(c) ^The item 'My child eats more when annoyed' loaded most highly onto the EUE factor (.55), but on theoretical grounds has provisionally been retained on the EOE scale, which is part of the newly developed factor OE.

^(d) ^The item 'My child eats more and more slowly during the course of a meal' loaded most highly onto the SR factor (.63), but has provisionally been retained on the SE factor, to provide better comparability with the original factor structure of the CEBQ.

^(e) ^The item 'My child is difficult to please with meals' also loaded onto the SR factor (.44).

### Reliability

Reliability coefficients (Cronbach's alphas) for the different scales of the instrument are presented in table 2. The coefficients ranged from .75 to .91 for the CEBQ subscales, which are all within acceptable ranges. The average item-total correlations, correcting for the contribution of the items to the scale, suggested adequate consistency of item content within the CEBQ subscales (.51 – .75) (table 2). Moreover, all corrected item-total correlations are considered 'good' (ranging from .39 to .84) [32].

**Table 2:** Factor structure and internal consistency of the CEBQ (*N *= 135)

	Number of factors with eigenvalue > 1	Percentage of variance Factor 1	Cronbach's alpha	Average corrected item-total correlation (range)
Food fussiness	1	70	.91	.75 (.64 – .84)
Enjoyment of food	1	57	.80	.60 (.39 – .67)
Overeating	2	42	.78	.51 (.39 – .64)
* Food responsiveness	*1*	*52*	*.72*	*.54 (.38 – .65)*
* Emotional overeating	*1*	*52*	*.67*	*.50 (.39 – .61)*
Satiety responsiveness	1	51	.76	.54 (.45 – .66)
Emotional undereating	1	63	.81	.62 (.54 – .72)
Desire to drink	1	67	.75	.59 (.44 – .69)
Slowness in eating	1	59	.76	.56 (.47 – .67)

*The items from two scales (FR and EOE) loaded onto the same factor, which we propose to name 'overeating'; when performing separate PCAs on the factor overeating, the two original factors, FR and EOE, were identified both with an eigenvalue > 1 (stated in italics). In this table, item 12 'My child is always asking for food', originally belonging the FR scale, was removed from this scale and incorporated in the factor EF.

### Age and gender differences

Independent samples t-tests were conducted to examine age and gender variations in children's eating behaviour (table 3). There were no statistically significant differences in parental responses regarding 6-year old children compared to parents of 7-year-olds. Significant gender differences were found. Boys scored higher on fussy eating (FF) than girls (mean 3.1 (SD 0.9) versus 2.6 (0.9), p = 0.000). Higher mean EOE values were found among boys (1.6 (0.5)) than among girls (1.3 (0.4)) (p = 0.003) and mean values for EF were higher for girls than for boys (girls 3.5 (0.6) versus boys 3.3 (0.7), p = 0.024).

**Table 3:** Mean (SD) of CEBQ subscale scores by gender (*N *= 135) and age group (*N *= 133*)

	Gender		Age group	
		
	Girls (*N *= 67)	Boys (*N *= 68)	6-years-old (*N *= 71)	7-years-old (*N *= 62)
Food responsiveness	1.8 (0.5)	2.0 (0.6)	1.8 (0.5)	2.0 (0.6)
Enjoyment of food	3.5 (0.6)	3.3 (0.7)	3.4 (0.7)	3.4 (0.7)
Emotional overeating	1.3 (0.4)	1.6 (0.5)	1.4 (0.5)	1.5 (0.5)
Desire to drink	2.3 (0.8)	2.5 (0.7)	2.3 (0.7)	2.5 (0.8)
Satiety responsiveness	2.8 (0.6)	2.8 (0.7)	2.8 (0.7)	2.8 (0.6)
Slowness in eating	2.6 (0.6)	2.8 (0.8)	2.8 (0.7)	2.6 (0.7)
Emotional undereating	2.2 (0.8)	2.3 (0.8)	2.2 (0.8)	2.3 (0.8)
Food fussiness	2.6 (0.9)	3.1 (0.9)	2.9 (0.9)	2.8 (0.9)

* Information on age was missing in 2 children

### Correlations between scales

The correlations between subscales of the CEBQ (table 4) indicate that the 'food approach' subscales (FR, EF, and EOE) and the 'food avoidant' subscales (SR, SE, EUE, and FF) tend to be positively inter-correlated. For the 'food approach' subscales, especially the FR-EF and FR-EOE correlations were found to have a large effect size. Moreover, a large correlation was found between the 'food avoidant' subscales SR and SE, whereas medium correlations were found for SR-FF and SE-FF. The 'food approach' subscales and the 'food avoidant' subscales were found to be negatively correlated. Large negative correlations were found for EF-SR, EF-SE, and EF-FF, whereas medium correlations exist for FR-SR and FR-SE. The only exception among these negative correlations was the medium-sized positive correlation between the 'food approach' EOE factor and the 'food avoidant' EUE factor. The correlations coefficients were compatible with the findings of Wardle et al. [7] and Viana et al. [6].

**Table 4:** Pearson's correlations between the CEBQ subscales (*N *= 135)

***CEBQ scales***	1 FR	2 EF	3 EOE	4 DD	5 SR	6 SE	7 EUE	8 FF
1 Food responsiveness (FR)	**-**							
2 Enjoyment of food (EF)	.50***	**-**						
3 Emotional overeating (EOE)	.54***	**.17**	**-**					
4 Desire to drink (DD)	.18*	.00	.16	-				
5 Satiety responsiveness (SR)	-.36***	-.59***	-.13	.09	**-**			
6 Slowness in eating (SE)	-.38***	-.53***	-.16	.07	.61***	**-**		
7 Emotional undereating (EUE)	.13	-.02	.41***	.05	.22*	.21*	**-**	
8 Food fussiness (FF)	-.18*	-.62***	.00	.15	.48***	.44***	**.14**	**-**

* p < .05; ** p < .01; *** p < .001 (two-sided); bold area upper-left corner: inter-correlations between 'food approach' subscales; bold area bottom-right corner: inter-correlations 'food avoidant' subscales

### Weight differences

A series of independent regression analyses was used to model each subscale of the CEBQ separately with child BMI z-scores entered as a continuous dependent variable, while correcting for potential confounding variables (child's gender and age, parental educational level, and parental employment status). In general, child BMI z-scores showed a linear increase with the 'food approach' subscales of the CEBQ (β 0.15 to 0.22), and a decrease with 'food avoidant' subscales (β -0.09 to -0.25) (table 5). Significant relationships were found for FR, EF (p < 0.05), and SR, SE (p < 0.01).

**Table 5:** Hierarchical linear regression analyses for BMI z-scores on CEBQ subscales (*N *= 135)

	Mean (SD)	Standardised β coefficient	95% CI for standardised β (lower bound – upper bound)	P-value
'Food approach' scales				
Food responsiveness	1.88 (0.56)	0.217	0.042 to 0.392	0.016
Enjoyment of food	3.40 (0.69)	0.207	0.025 to 0.389	0.027
Emotional overeating	1.47 (0.48)	0.145	- 0.036 to 0.326	0.118
'Food avoidant' scales				
Satiety responsiveness	2.77 (0.65)	- 0.240	- 0.409 to -0.071	0.006
Slowness in eating	2.73 (0.75)	- 0.248	- 0.421 to -0.075	0.006
Emotional undereating	2.27 (0.79)	- 0.088	- 0.269 to 0.093	0.344
Food fussiness	2.84 (0.90)	- 0.103	- 0.284 to 0.078	0.270

Child gender and age, maternal and paternal education level, and maternal and paternal employment status were forced into the models before adding each of the CEBQ scales separately. Standardised β coefficients (p-values) were 0.033 (p = 0.715), 0.030 (p = 0.734), -0.021 (p = 0.852), -0.051 (p = 0.658), 0.122 (p = 0.190) and 0.029 (p = 0.752) for the control variables respectively.

**Figure 1:**
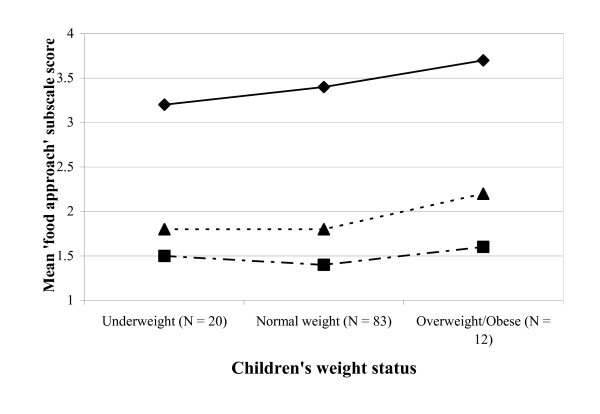
**Mean 'food approach' scores by Body Mass Index category**. Children's Eating Behaviour Questionnaire subscales: - - - - - - - - - , food responsiveness;   - — - — - — , emotional overeating; ———–— , enjoyment of food

**Figure 2:**
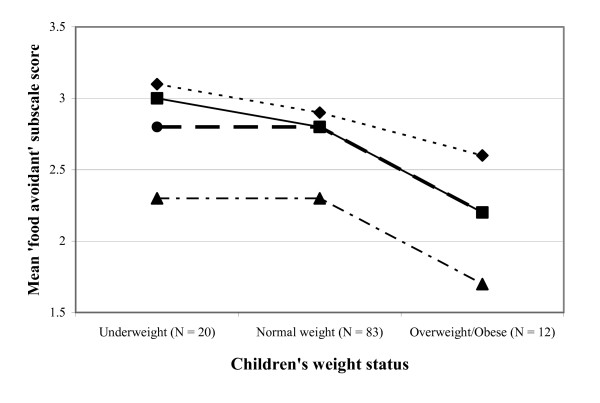
**Mean 'food avoidant' scores by Body Mass Index category**. Children's Eating Behaviour Questionnaire subscales: — — — — — , satiety responsiveness;  ———–— , slowness in eating; — - — - — - — , emotional undereating; - - - - - - - - -  , food fussiness

The results regarding differences in scale scores across child BMI groups (one-way analysis of variance) are graphically displayed in figures 1 and 2, illustrating mean 'food approach' and mean 'food avoidant' scores by weight status category. Significant differences between weight categories were found for the factors SR (F = 3.69, p < 0.05) and SE (F = 3.86, p < 0.05). Normal-weight subjects scored significantly higher on the SR scale than the overweight/obese subjects (mean score (SD) normal-weight subjects 2.8 (0.7) versus overweight/obese subjects 2.3 (0.7), p = 0.02). For the SE scale significant differences were found between underweight and overweight/obese children, with underweight subjects scoring higher (mean (SD) 3.0 (0.5) versus 2.2 (0.5), p = 0.02).

## Discussion

The present study showed good psychometric properties of the Dutch translation of the CEBQ in terms of factor structure, internal reliability and correlations between subscales corresponding very closely to the original study [7] and a recent Portuguese validation study of the CEBQ [6]. In our sample of 6- and 7-year-old Dutch children a seven-factor structure was the best interpretable solution, which explained 62.8% of the variance. In parallel with earlier studies [6,7], the original eight-factor structure could not perfectly be replicated. In comparison to the original factor structure [7], the scales of FR and EOE were clustered together in the present Dutch sample to ascertain the psychometric properties of this study. The FR and EOE scales were highly correlated, and combining them into one scale ('overeating') increased the internal consistency coefficient. However, caution is needed when combining those two scales, since they may differentiate in older age groups and it should be noted that the original FR and EOE scales were revealed in a separate Principal Components Analysis on the combined scale.

Cross-sectional associations between the mean scale scores and BMI showed that overweight children displayed weaker satiety responses and stronger appetite responses to food compared to their leaner counterparts. This result is in line with the Portuguese study [6]. In addition, overweight children appeared to apply poorer eating regulatory mechanisms and to have an increased eating rate compared to normal-weight children. The positive association of the scales FR and EF with child's BMI z-score is consistent with research demonstrating that children with a higher BMI are highly responsive to environmental food cues [e.g., [5-7,13,28]]. SR and SE were inversely associated with child BMI z-score similar to the recently published study of Carnell and Wardle [13] and Viana et al. [6]. In the current study, EUE and FF were found to have the weakest associations with the BMI z-score. This result parallels those reported by Viana and colleagues [6], suggesting that these eating behaviours are less strongly related to child weight. Moreover, this low non-significant association of fussiness with the child's BMI resembled findings of other studies [23,25,26]. More studies are needed applying the CEBQ cross-culturally to confirm these findings.

A recently published study in the Netherlands [9] suggested that emotional undereating was a more salient dimension for young children than emotional overeating. Young children react to emotional distress (loss of appetite when feeling e.g. upset or anxious) with a biologically natural response, which includes a reduction of gut activity thereby reducing children's food intake [34]. Indeed, consistent with findings from previous research [7,9], we found a low mean scale score on the EOE scale, confirming that eating in response to emotional stressors is quite abnormal in young children. In addition, our results support the psychosomatic theory [35,36], which posits that people overeat as a way of coping with emotional stressors based on experiences learned early in life. Our study indicates that this learned response to distress is not yet well-established in children as young as 7 years of age (see also [15]).

In contrast to the studies of Wardle and colleagues [7] and Ashcroft and colleagues [15], no age effects were found for the CEBQ subscales. This may well be due to the narrow age range in our study (29 months), whereas the age range in the study of Wardle et al. [7] and Ashcroft et al. [15] was at least 4 and 6 years respectively. Similar to the findings reported by Wardle et al. [7], we found gender differences for FF, with boys scoring higher on fussy eating than girls. However, we also found significant differences for EOE (boys emotionally overeat more often than girls) and EF (girls enjoying food more often than boys). Since many differences in eating behaviours are detected during the teenage years among boys and girls, it would be advisable to track the development of gender differences in eating styles from early childhood onwards. Additionally, more research is needed to assess the exact role of gender in child eating behaviours, possibly in interaction with parental feeding styles [37].

Recently, evidence has been found regarding heritability of certain appetitive traits known to be related to the development of obesity. Carnell and colleagues [38] found evidence for a strong genetic influence of satiety and food cue responsiveness in children. In addition, Wardle et al. [39] have shown that genetic variants could contribute to lower sensitivity to satiety cues. These genetic influences on children's appetite responses indicate the importance of identifying high-risk children in early childhood, since they are more likely to overeat when encountering obesogenic environments.

The present study has several limitations that should be acknowledged. First, factor-analytic procedures have to be repeated on a larger sample of Dutch 6- and 7-year olds to replicate our findings. In addition, considering the small sample size, confirmation regarding the associations between various eating styles and BMI in Dutch children age 6 and 7 is needed. Second, the response rate was relatively low (mean 41.9%) and families with lower levels of education were relatively underrepresented in the current study. Another limitation was that the children's weight and height were parentally reported and not directly measured. Compared with measured weight and height, parents of 4-year-old children have been shown to slightly underestimate their children's weight and overestimate height, especially if their child was overweight or obese, whereas parents of underweight children tended to overestimate weight [40]. Hence, our study reported slightly lower percentages of overweight/obesity (10.4%) compared to the Dutch reference population of children aged 6 and 7 (2002–2004: ranging from 12.5% to 18.7%) [41]. It is likely that the present study yielded underestimates of associations between the instruments' scale scores and BMI, because of the parental reported nature of this study. In addition, there is a potential bias if parents who did not complete the questions regarding their children's weight and height had responded differently to distinct subscales than parents who completed those questions. However, except for DD, with slightly higher DD scores in those with missing height and weight data than in those with data present, no differences on any of the subscales were present. Finally, due to the cross-sectional nature of the study, inferences regarding causality cannot be made. Longitudinal and experimental study designs are needed to strengthen inferences, and assess the exact role of children's eating behaviours in the aetiology of obesity.

## Conclusion

This study is the first to evaluate the factor structure of the CEBQ in a Dutch population among parents of children aged 6 or 7. In summary, the findings of the present study suggest that the instrument is valuable for identifying specific eating styles, which can be seen as important and modifiable determinants implicated in the development and maintenance of overweight and obesity. The identification of such variables is a prerequisite to gain insight into the behavioural pathways to obesity, and subsequently for the development of evidence-based intervention programs to prevent obesity in young children. Further longitudinal studies are needed to assess the role of eating behaviours in the development of obesity during childhood and into adulthood.

## Competing interests

The authors declare that they have no competing interests.

## Authors' contributions

All authors contributed to the design of the study. ES conceptualised the study, performed recruitment of study participants and data collection, conducted the statistical analyses, and drafted the manuscript. ES and SK translated the instrument into the Dutch language. SK and CT both critically reviewed the analytical procedures used and participated in the interpretation of the results. SK and CT reviewed draft versions of the manuscript and provided critical feedback. All authors have made a significant contribution to this manuscript, and all authors read and approved the final manuscript.
